# Medical education advances and innovations: A silver lining during the COVID-19 pandemic

**DOI:** 10.36834/cmej.70926

**Published:** 2020-12-07

**Authors:** Nishila Mehta, Céline Sayed, Rishi Sharma, Victor Do

**Affiliations:** 1 Faculty of Medicine, University of Toronto, Ontario, Canada; 2 Faculty of Medicine, University of Ottawa, Ontario, Canada; 3 Department of Internal Medicine, McMaster University, Ontario, Canada; 4 Hospital for Sick Children, University of Toronto, Ontario, Canada

## Abstract

The COVID-19 pandemic has disrupted healthcare processes substantially including medical education, necessitating several changes along the spectrum of medical training. While this crisis presents major challenges to medical education, it is also an immense opportunity for innovation. In this commentary, Canadian medical students cast a spotlight on four domains of Canadian medical education which have seen substantial changes during the COVID-19 pandemic: medical school admissions, pre-clerkship content delivery, virtual care and telemedicine curricula, and the residency matching process. Using the 10 recommendations noted in the Association of Faculties of Medicine of Canada (AFMC) 2010 Future of Medical Education in Canada report as a guiding framework, we discuss why these changes represent key steps forward that should be preserved in medical education beyond the pandemic, and advocate for a continuous quality improvement approach to evaluate and implement these innovations.

## Abstract (fr)

La pandémie COVID-19 a considérablement perturbé les processus de soins de santé, y compris l’éducation médicale, ce qui a nécessité plusieurs changements dans le spectre de la formation médicale. Si cette crise pose des défis majeurs en éducation médicale, elle constitue également une immense opportunité d'innovation. Dans ce commentaire, les étudiants en médecine canadiens mettent en lumière quatre domaines de l’éducation médicale canadienne qui ont connu des changements substantiels durant la pandémie COVID-19 : les admissions dans les facultés de médecine, enseignement des cours au pré-externat, les cursus de soins virtuels et de télémédecine, et le processus de jumelage des résidents. En utilisant les 10 recommandations mentionnées dans le rapport 2010 sur l'avenir de l'enseignement médical au Canada de l'Association des facultés de médecine du Canada (AFMC) comme cadre d'orientation, nous expliquons pourquoi ces changements représentent des étapes clés qui devraient être préservées en éducation médicale au-delà de la pandémie, et nous préconisons une approche d'amélioration continue de la qualité pour évaluer et mettre en œuvre ces innovations.

The COVID-19 pandemic has substantially disrupted all aspects of healthcare including medical education. Medical educators have had to adapt to a ‘new normal’, swiftly innovating both school-specific and national activities.^1^ Previous adaptations due to public health crises have contributed to permanent innovation in medical education, such as online problem based learning.^2^ However, there have also been many missed opportunities for improvements in medical education during prior public health crises.^3^ It is therefore paramount that despite the challenges posed by the COVID-19 pandemic, the present opportunity for innovation in Canadian medical education is seized.

In 2010, the Future of Medical Education in Canada (FMEC) report, commissioned by the Association of Faculties of Medicine of Canada (AFMC), outlined 10 recommendations for undergraduate medical education, with five additional enabling recommendations.^4^ Using these recommendations as a guiding framework, we provide an overview of medical education advances that have been implemented in response to the COVID-19 pandemic which we, as a medical education community in Canada, should work to preserve.

## Medical school admissions processes

Recommendation II from the FMEC project is to *Enhance Admissions Processes* to accurately assess for ‘values and personal characteristics of future physicians, and increase diversity in the physician workforce.’^4^

A well-recognized medical school admissions barrier is financial costs, including flights and accommodation for interviews.^5^ During the 2019-2020 admissions cycle, travel restrictions necessitated the transition to online interviews at several medical schools, increasing the financial accessibility of interviews. Certain schools such as Queen’s University implemented two-stage interviews: applicants successful in a virtual interview were invited to the next one. Moving forward, continuing with a “two-stage” interview model could substantially reduce the overall applicant costs of interviewing at medical schools.

Ongoing COVID-19 impacts are leading to more admissions-related changes for the 2020-2021 cycle, including admissions GPA calculation methods, Medical College Admission Test requirements, and increased reliance on computerized assessments such as the Computer-Based Assessment for Sampling Personal Characteristics (CASPer).^6,7^ Medical schools should study the impacts on the composition of medical school enrollees resulting from these changes, and consider long-term implementation of changes which move us towards greater diversity in our student populations.

## Delivery of content virtually in pre-clerkship years

During pre-clerkship, students learn foundations of medicine largely through large group lectures. Increasingly, pre-clerkship students are showing preference for remote and flexible learning.^8^

Due to the pandemic, medical schools rapidly transitioned to online learning to deliver pre-clerkship curriculum. The literature suggests this is as effective as, and in some contexts, more effective than in-person learning.^9,10^ This is the ideal time for medical schools to effectively integrate technology into curriculum delivery, aligning themselves with FMEC enabling recommendation D: *Improve the Use of Technology* to maximize the benefits of both online and offline learning.^4^ Harnessing technology can enable schools to move towards innovative models of medical education such as “flipped classroom” teaching, which has been endorsed as better preparing learners for today’s digitally empowered medical practice.^11^ Importantly, adequate training and support of faculty members should be undertaken during transitions to online learning, as recognized by FMEC enabling recommendation E: *Enhance Faculty Development*.^4^

## Virtual care & telemedicine in undergraduate medical education

At the outset of the pandemic, clinical rotations for senior medical students across Canada were suspended.^1^ Students have now returned to significantly changed clerkship rotations. In addition to new safety protocols and sometimes lower patient volumes, a major transition has been an increase in virtual care. Consequently, students’ return to the clinical environment has been accompanied by experiential opportunities in virtual care, which prior to the COVID-19 pandemic, was largely absent in Canadian medical education. With widespread calls for continuing advances in virtual care that occurred during the pandemic, we must also instill virtual care and telemedicine as permanent components of medical education.^12^

This aligns with FMEC recommendation VI to *Diversify Learning Contexts*.^4^ In addition, enabling recommendation E, *Enhance Faculty Development*, is necessary for curricular integration of virtual care, as clinician-teachers should be familiarized with best practices in virtual care teaching.

## Residency matching processes

The Canadian residency matching process is undergoing substantial changes in the 2020-2021 cycle, including a national transition to virtual residency interviews and cancellation of visiting electives.

The cancellation of visiting electives has sparked concerted efforts to reduce the emphasis of on-site elective completion in residency application evaluation. Completing an on-site elective has been an unwritten ‘requirement’ of matching to certain competitive residency programs, and this ‘requirement’ has been linked to elective selection behaviours driving an increasing rate of unmatched Canadian medical graduates.^13^ As FMEC Recommendation V strives to *Address the Hidden Curriculum*, and unwritten yet expected elective ‘requirements’ are a part of this curriculum, this is an opportunity to promote a more transparent, equitable match process.^4^

Further, virtual interviews for the R1 residency match have long been advocated by many learners due to the financial, environmental, and personal toll of in-person interviews. Serious consideration should be given to preserving virtual interviews, which through reducing financial barriers to interviewing have the possibility to improve equity during the R1 match process, ensuring this transition does not adversely impact students’ ability to match.

Finally, both the cancellation of electives and in-person interviews have impacted the opportunity for medical students to explore residency programs outside of their medical school. Programs now have increased burden to promote themselves to medical students through online modalities. These potential initiatives should be sustained in future years to increase accessibility to exploring residency programs and assist in career exploration.

## Conclusion

The COVID-19 pandemic has stimulated and accelerated many innovations in undergraduate medical education in Canada and across the globe. We must be vigilant to avoid automatically returning to the previous “normal” post-pandemic reality, which may have included practices that were less effective or desirable.

Innovations that we have adopted during the COVID-19 pandemic should be thoroughly studied and evaluated using a continuous quality improvement framework. The 2010 FMEC project initiated by the AFMC laid out several recommendations for MD education that have yet to be fully realized- now is the time to renew the collective commitment to FMEC goals. Educators should be brought together to reimagine, plan and implement a new system of medical education that incorporates, builds on and refines innovations implemented during the COVID-19 pandemic.
